# An Analysis of Regional Blocks During Enzymatic Debridement of Burns

**DOI:** 10.1093/jbcr/iraf084

**Published:** 2025-05-14

**Authors:** Zoey Chasen, Justin Suarez, Alexander Kurjatko

**Affiliations:** Department of Pharmaceutical Care, University of Iowa, Iowa City, IA, United States; Department of Pharmaceutical Care, University of Iowa, Iowa City, IA, United States; Department of Surgery, Acute Care Surgery Division, University of Iowa, Iowa City, IA, United States

**Keywords:** anacaulase, pain, regional blocks, enzymatic debridement

## Abstract

Anacaulase is a mixture of enzymes used for the breakdown of eschar in patients with deep partial- or full-thickness burns up to 20% of the body’s surface area. European consensus guidelines recommend regional anesthesia of an isolated extremity undergoing enzymatic debridement, but no North American consensus has been reached. Our practice has shifted from a multimodal pain strategy to utilize regional blocks prior to the application of anacaulase. The purpose of this study was to determine whether the introduction of regional blocks improved pain control in patients receiving anacaulase therapy. This is a single-center retrospective comparative study evaluating all patients who received anacaulase from July 2016 to July 2024. Continuous data was evaluated via the Wilcoxon rank sum test and categorical data was evaluated via the chi-square test of independence. In the prespecified period, 47 patients received anacaulase and were included for analysis. Of the 47 patients, 24 (51.1%) underwent regional block. There were no significant differences in baseline characteristics except lower baseline opioid requirements in the regional block group. Pain scores during anacaulase application were significantly lower in patients that received regional blocks (4.5 vs 7, *P* = .003). There was no difference in change in opioid requirements or requirement of adjunct medications except for dexmedetomidine requirements (0% vs 78.3%, *P* < .001). Our data suggest that regional blocks were associated with lower pain scores in burn patients receiving anacaulase therapy but did not result in a change in opioid requirements from baseline. Regional blocks may be an effective strategy in this population to reduce pain from enzymatic debridement, but prospective studies would be warranted.

## INTRODUCTION

Acute pain from burn injuries is a source of patient suffering, linked to chronic pain and stress-related disorders.^1^ An interview of burn patients demonstrated variable experiences, but a desire to receive more education on pain expectations.^2^ The assessment and management of pain in burn patients is an important aspect of their care, with effect on short-term and long-term outcomes.^3^ A combination of analgesic and nonpharmacologic therapies is a primary strategy in management of pain, with consideration to the complex interplay of pathophysiology, psychosocial, and biochemical changes seen in burns.^4^

Meanwhile, novel therapies are emerging in the field of burn surgery that show potential in decreasing the surgical demands these patients face. Among these therapies is the use of anacaulase, which is a mixture of enzymes used for breakdown of eschars in patients with deep partial- or full-thickness burns up to 20% of the body’s surface area.^5^ While anacaulase remains on the skin for hours, patients may experience significant pain. A European consensus guideline recommends regional anesthesia of an isolated extremity undergoing enzymatic debridement, while a Spanish consensus presents regional anesthesia as an alternative to intravenous analgesia.^6,7^ In North America, no such consensus guidelines have yet been reached. This is likely due to the fact that the United States Food and Drug Administration (US FDA) only awarded approval for anacaulase for its use in burn-injured populations in January 2023.

Our burn center was involved in trials of anacaulase prior to US FDA approval. Through our burn center’s use of anacaulase therapy, our pain management practices have evolved. Whereas our initial strategy focused on multimodal pain regimens with opiates and anxiolytics, we began incorporating the use of regional blocks prior to application of anacaulase. Given the invasive nature and inherent risks of regional blocks, it is essential to ensure that the potential benefits of improved pain control outweigh those risks. The purpose of this study is to compare the pain experiences between patients undergoing anacaulase therapy who received multimodal pain regimens with and without regional nerve blocks. We hypothesized that the pain scores and morphine milligram equivalents (MME) are decreased among the cohort that incorporated treatment with regional nerve blocks.

## METHODS

### Ethical statement

Our Institutional Review Board approved this retrospective study (IRB # 202408047). A waiver of consent was approved for all subjects.

### Study design

Our institution’s burn registry was queried retrospectively to identify all burn patients aged 18 and older admitted from July 31, 2016 to July 31, 2024 who received topical enzymatic debridement therapy with anacaulase. Patients without pain scores recorded during the prespecified timepoint were excluded from primary endpoint analysis. The study follows the Strengthening the Reporting of Observational Studies in Epidemiology (STROBE) reporting guidelines for cohort studies.^8^

### Institutional practice

With guidance from the DETECT trial protocols,^9^ adult patients with deep partial- or full-thickness burns involving 3% to 30% TBSA are considered for use of enzymatic debridement within the first 84 h of injury. Patients with circumferential burns of the limbs, infected burns, inhalation injury, pregnancy, and a major comorbidity are excluded from consideration. Blisters from the burned tissue are removed and the burn tissue is soaked in an antibacterial solution for at least 2 h. Anacaulase is then applied to the wound with a dose of 2-g sterile powder mixed with 20-g sterile gel per 1% TBSA burn. A barrier of petroleum gel is applied at the edges of the burn, and the wound is covered with occlusive dressing for 4 h. After 4 h, the dressings are removed, and the enzyme and burn eschar is gently removed with a wooden tongue depressor. The wounds are then soaked in an antibacterial solution for 2 h, and then cleaned before placing in standard antibacterial-coated wound dressings.

### Data collection

Demographics (age, sex, race, ethnicity), injury information (TBSA), hospital course (hospital length of stay, hospital cost) were obtained from our institution’s Burn Registry. Medical records were reviewed to identify patients who met our inclusion criteria. The following variables were also collected from medical records: Opioid use prior to admission, time of anacaulase administration, dose of anacaulase, pain scores at 4-h intervals, time of regional anesthesia, type of regional anesthesia provided, medication used in regional anesthesia, opiate medications given in 4-h intervals, adjunct medications provided within 4-h intervals of anacaulase administration (dexmedetomidine, acetaminophen, midazolam, gabapentin, ibuprofen).

### Statistical analysis

Normality was assessed using the Shapiro–Wilk test for all continuous variables. All nonnormally distributed continuous variables are presented as median and interquartile range. Chi-square test or Fisher’s exact test was used to compare categorical variables. Continuous data was evaluated via the Wilcoxon rank sum test. A 2-sided *P*-value <.05 was considered significant.

Baseline measurements for pain scores and MME were calculated using values from the 4 h prior to anacaulase administration. If there was a baseline difference between groups for pain scores or MME, we planned to analyze the change from baseline, calculated as the difference between the baseline measurement and the measurement during anacaulase administration.

## RESULTS

### Patient characteristics

Patient characteristics, injury information, hospital length of stay, hospital cost, individuals with prior opioid use, anacaulase dose, baseline pain scores and baseline MME 4 h prior to enzymatic debridement are presented in Table 1. There were 47 patients who underwent enzymatic debridement with anacaulase during the study period. Of these, 24 (51.1%) received regional anesthesia in addition to multimodal pain management. Thirty-four (72.3%) patients were male, 39 (83.0%) were Caucasian, 4 (8.5%) were Hispanic, 2 (4.2%) were African American, and 1 (2.1%) was Pacific Islander. There were no statistical differences in age, sex, ethnicity, or TBSA between groups. There were no statistical differences in patients with opioid use prior to admission between groups. The dose of anacaulase was not statistically different between groups (22.0 g [7.5, 153.8] vs 27.0 g [18.5, 55.0]; *P* = .798). The length of time from admission to enzymatic debridement was lower among the cohort that received regional anesthesia (1.6 days [1.3, 1.9] vs 2.0 days [1.6, 2.8]; *P* = .069) though this did not reach statistical significance. The pain scores 4 h prior to enzymatic debridement were not statistically different between groups (4.0 [3.0, 6.0] vs 4.5 [3.0, 6.0]; *P* = .525). The MME 4 h prior to enzymatic debridement was significantly lower (20.0 [9.8, 27.6] vs 33.6 [26.8, 45.0]; *P* < .001) for those who underwent regional anesthesia.

**Table 1.** Baseline Characteristics

		Regional blocks (*n* = 24)	No regional blocks (*n* = 23)	*P*-value
Age, years		35.5 [23, 59.8]	39.0 [26.5, 57.5]	.670
Male		16 (66.7)	18 (78.3)	.374
Ethnicity	Caucasian	19 (79.2)	20 (87)	.570
	Hispanic	2 (8.3)	2 (8.7)	
	African American	2 (8.3)	0 (0)	
	Pacific Islander	0 (0)	1 (4.3)	
	Unknown	1 (4.2)	0 (0)	
Prior to admission opioid use		1 (4.2)	3 (13)	.57
Burn total body surface area %		8.5 [4.8, 14.6]	10.6 [6.5, 14.5]	.322
Anacaulase dose, grams		22.0 [7.5, 153.8]	27.0 [18.5, 55.0]	.798
Time from admit to anacaulase administration, days		1.6 [1.3, 1.9]	2.0 [1.6, 2.8]	.069
Time from regional block to anacaulase administration, minutes		21.0 [−8.3, 62.8]	–	–
Regional block type	Single-stick	17 (70.8)	–	–
	Continuous	5 (20.8)		
	Both	2 (8.3)		
Regional block medication	Ropivacaine	17 (70.8)	–	–
	Bupivacaine	5 (20.8)		
	Bupivacaine and ropivacaine	1 (4.2)		
	Ropivacaine and mepivacaine	1 (4.2)		
Duration of continuous catheter anesthetic, hours		24.1 [20.7, 37.1]	-	–
Pain scores 4-h baseline		4.0 [3.0, 6.0]^a^	4.5 [3.0, 6.9]^b^	.525
Morphine milligram equivalent 4-h baseline		20.0 [9.8, 27.6]	33.6 [26.8, 45.0]	<.001
Hospital cost, dollars		79 601 [29 509, 171 972]^c^	71 284 [35 437, 139 946]	.711
Hospital length of stay, days		6.2 [3.6, 12.9]	9.9 [5.3, 14.7]	.295

Data presented as *n* (%) or median [interquartile range].

^a^ *N* = 21 due to missing values.

^b^ *N* = 22 due to missing values.

^c^ *N* = 23 due to missing values.

### Primary and secondary endpoints

Pain scores following anacaulase administration are shown in Table 2. Pain scores during anacaulase administration were statistically lower in the group receiving regional anesthesia (4.5 [2.6, 5.8] vs 7.0 [6.0, 8.8]; *P* = .003). Morphine milligram equivalents during anacaulase administration were significantly lower among patients who underwent regional anesthesia (22.5 [3.6, 30.0] vs 33 [23.5, 36.7]; *P* = .020), but the change in MME from baseline was not significantly different between groups (0 [−12.8, 10.0] vs 3.6 [−12.4, 11.5]; *P* = .205). There were no differences in adjunct medications used between groups, with the exception of dexmedetomidine, which was significantly lower among patients receiving regional anesthesia (0 [0%] vs 18 [78.3%]; *P* < .001).

**Table 2.** Primary Endpoint Results

		Regional blocks (*n* = 24)	No regional blocks (*n* = 23)	*P*-value
Pain scores during 4-h anacaulase administration period		4.5 [2.6, 5.8]^a^	7.0 [6.0, 8.8]^b^	.003
Morphine milligram equivalents during 4-h anacaulase administration period		22.5 [3.6, 30]	33 [24.5, 38]	.020
Change in morphine milligram equivalents		0 [−12.8, 10.0]	3.6 [−12.4, 11.5]	.205
Adjunct medications required during 4-h anacaulase administration period	Dexmedetomidine	0 (0)	18 (78.3)	<.001
	Acetaminophen	23 (95.8)	23 (100)	>.999
	Midazolam	20 (83.3)	17 (73.9)	.494
	Gabapentin	0 (0)	1 (4.3)	.489
	Ibuprofen	0 (0)	1 (4.3)	.489

Data presented as *n* (%) or median [interquartile range].

^a^ *N* = 18 due to missing values.

^b^ *N* = 19 due to missing values.

## DISCUSSION

Enzymatically controlled debridement of burned tissue has emerged as a relatively new supplement to surgical debridement. A multicenter randomized controlled trial recently demonstrated decreased surgical excision needs, lower blood loss, and earlier eschar removal with enzymatic debridement compared to the standard of care.^9^ The use of enzymatic debridement may contribute to reduction in overall cost of burn care.^10^ As the role for enzymatic debridement expands for burn populations,^11–13^ the importance of addressing pain needs during application increases.

The pain associated with enzymatic wound debridement had initially necessitated the eschar removal be performed under general anesthesia. In 2018, Galeiras et al. found that procedural sedation and analgesia allowed for enzymatic debridement without requiring invasive mechanical ventilation, though only 2 patients received local or regional anesthesia.^14^ In 2020, Arkoulis et al. performed a study on 20 adults undergoing enzymatic debridement and utilized anesthetist-led analgesia in all patients evaluated.^15^ In 2022, Claes et al. showed that regional anesthesia facilitated the use of enzymatic debridement without general anesthesia.^16^ A questionnaire that retrospectively assessed patient satisfaction after enzymatic debridement revealed that enzymatic debridement could be performed satisfactorily without general anesthesia, utilizing analgosedation, regional anesthesia, or local anesthesia.^17^ In 2023, Buta et al. noted similar pain scores between patients receiving a regional block when compared to patients receiving local anesthesia or conscious sedation.^18^ Our study adds to the literature, with no patients requiring general anesthesia, and improved pain scores with regional anesthesia. Beyond 4 h, however, the improvement in pain scores no longer has statistical significance. Although we demonstrate a lower amount of MME during enzymatic debridement, the change in MME use from baseline does not reach statistical significance.

Regional anesthesia is an adjunctive pain control strategy involving the administration of local anesthetic drugs placed centrally in the epidural or intrathecal spaces or near major nerves. Peripheral nerve blocks can be performed as a single-shot or continuous catheter-directed infusion. Although there is a large amount of evidence to support the use of regional anesthesia in the perioperative acute pain literature, there is limited data in its use for burn pain.^19^ A retrospective analysis on continuous catheter-directed peripheral nerve block demonstrated the ability to decrease the use of general anesthesia for debridement of burn wounds, with an infection rate similarly as low as other surgical populations.^20^ Our study notes no complications related to catheter placement for regional anesthesia. A meta-analysis on the use of regional anesthesia for skin graft donor sites demonstrated it to be a cost-effective approach that decreased narcotic consumption, decreased side effects, and may result in shorter hospital days.^21^ Additionally, reducing the use dexmedetomidine for sedation purposes has the potential to decrease overall costs.^22^ We note no difference in costs between the 2 pain strategy cohorts, suggesting that utilization of regional blocks would not significantly raise hospital costs for patients.

A multiprofessional expert panel of plastic surgeons and burn specialists from twelve European centers unanimously agreed that regional anesthesia is recommended for enzymatic debridement of burn isolated to an upper or lower extremity.^6^ The panel endorsed either the use of continuous regional anesthesia via a catheter, or a single-shot with long-lasting locally acting anesthesia. Similarly, an agreement was reached in a Spanish multidisciplinary consensus that locoregional anesthesia is an alternative to intravenous analgesia in patients with burns in the extremities, and that enzymatic debridement does not routinely require general anesthesia.^7^ In North America, there has not yet been any established consensus statement on the pain management during enzymatic debridement. To our knowledge, our study is the first to compare the effectiveness of multimodal pain regimens with and without regional anesthesia in the setting of enzymatic debridement of burns.

This study has many limitations. As a single-center retrospective study with a small sample size, causality cannot be established, and findings may not be generalizable. Patient-reported numerical pain scores are a subjective method of measuring pain and may be variable between patients. While it did not reach significance, the time from admission to enzymatic debridement trended higher in patients who did not undergo regional anesthesia. There has not yet been any literature assessing a difference in pain experiences based on the length of time to enzymatic debridement in burns. We noted a baseline difference in MME requirements prior to enzymatic debridement between groups, which implies the possibility of confounding variables effecting the patient’s perception of pain. Because the group receiving pain catheters had a lower baseline MME requirement prior to therapy, an unforeseen selection bias of a more pain-tolerant cohort may have contributed to the same group requiring less MME during and after therapy. The involvement of specialty pain services for patients receiving regional anesthesia may have exposed that group to better communication regarding expectations, which has been shown to result in lower levels of pain intensity and may be a confounder to our findings.^23^ Additionally, it is possible that the involvement of specialty pain services led to disincentive among the primary burn services and nursing staff to pyramid pain medications ahead of anacaulase application. However, our study demonstrates a subjective benefit in utilizing regional anesthesia among patients undergoing enzymatic debridement on a burned extremity. Prospective studies are warranted to further determine the role of regional anesthesia in controlling pain during enzymatic debridement.

## CONCLUSION

Our data suggest that adding regional anesthesia to a multimodal pain regimen improved pain scores among burn patients undergoing enzymatic debridement, compared to multimodal pain therapy without regional anesthesia. Although regional anesthesia was associated with a lower MME requirement during enzymatic debridement, there was no change in MME requirements from baseline. Regional anesthesia significantly reduced the need for analgesia with dexmedetomidine. Regional blocks may be an effective strategy to control pain during enzymatic debridement.
